# Mechanical Properties of Gas Main Steels after Long-Term Operation and Peculiarities of Their Fracture Surface Morphology

**DOI:** 10.3390/ma12030491

**Published:** 2019-02-05

**Authors:** Volodymyr Hutsaylyuk, Pavlo Maruschak, Ihor Konovalenko, Sergey Panin, Roman Bishchak, Mykola Chausov

**Affiliations:** 1 Department of Machine Design, Military University of Technology, Gen. S. Kaliskiego str. 2, 00-908 Warsaw, Poland; 2 Department of Industrial Automation, Ternopil National Ivan Pul’uj Technical University, Rus’ka str. 56, 46001 Ternopil, Ukraine; maruschak.tu.edu@gmail.com (P.M.); icxxan@gmail.com (I.K.); 3 Institute of Strength Physics and Materials Science of Siberian Branch of Russian Academy of Sciences, 634055 Tomsk, Russia; svp@ispms.ru; 4 Department of Welding, Ivano-Frankivsk National Technical University of Oil and Gas, Ivano-Frankivsk, Karpatska str. 15, 76019 Ivano-Frankivsk, Ukraine; bishchakr@gmail.com; 5 Department of Mechanics, National University of Life and Environmental Sciences of Ukraine, Heroiv Oborony str.15, 03041 Kyiv, Ukraine; m.g.chausov@gmail.com

**Keywords:** gas mains steel, failure, static cracking resistance, long-term operation

## Abstract

Regularities of steel structure degradation of the “Novopskov-Aksay-Mozdok” gas main pipelines (Nevinnomysskaya CS) as well as the “Gorky-Center” pipelines (Gavrilovskaya CS) were studied. The revealed peculiarities of their degradation after long-term operation are suggested to be treated as a particular case of the damage accumulation classification (scheme) proposed by prof. H.M. Nykyforchyn. It is shown that the fracture surface consists of sections of ductile separation and localized zones of micro-spalling. The presence of the latter testifies to the hydrogen-induced embrittlement effect. However, the steels under investigation possess sufficiently high levels of the mechanical properties required for their further safe exploitation, both in terms of durability and cracking resistance.

## 1. Introduction

It is known that 17MnSi and 17Mn1Si steels were widely used for manufacturing gas mains over the territory of the former Soviet Union [1,2,3]. Conventionally, main gas pipes were insulated with mastic and film protective coatings. Nowadays, the run-time of these gas pipelines exceeds 30 years, while in some cases it reaches even 50 years of operation [3,4,5,6,7,8].

Gas mains with the diameter of 1420 mm are insulated with film coatings, while gas pipes of the smaller diameter of 1020 mm are protected by bituminous insulations. However, the design specified that the durability of such coatings does not exceed 15 years. Regardless of the permanent maintenance of corrosion protection stations, the degradation of insulating coating often results in the corrosion of pipes [9,10]. In addition, various degradation processes such as, (I) micro- and macrodeformation, (II) strain aging, (III) accumulation of structural damages, and (IV) nucleation of microcracks occur in pipe steels. When in contact with corrosive media, hydrogenation of the pipe wall can be initiated [11]. These processes possess a stage pattern, however, they can significantly vary in various climatic zones. In our previous studies, the degradation of pipe steels exploited in Ukraine and Yakutia (Russia) [8,12,13] was investigated. The regularities of their mechanical properties degradation were summarized in a set of schemes. One of which was initially proposed in the papers of Prof. H.M. Nykyforchyn [8].

The detailed study including a physical–mechanical interpretation of structure and properties degradation due to long-term exploitation of gas main steels, make it possible to predict their integrity and estimate the probability of their failure [14,15]. To ensure the reliability of such predictions, it is of importance to use modern techniques for investigating the structure and the mechanical properties. In this sense, the method of complete stress–strain diagrams [16] with its clear physical background and simple mechanical realization under static and dynamic loading is promising. It was shown that this method allows one to ensure appropriate testing conditions that guarantee the “stability” of deformation and fracture processes at different stages, even at the stage of macroscale failure.

The aim of this study is to establish general regularities of structure and properties degradation of the gas main steels after long-term operation (30–39 years).

## 2. Experimental Technique

Fragments of the gas mains exploited in the Russian Federation over a long period of time (30–39 years) were studied, see Table 1.

Cylindrical shape specimens with a diameter 5 mm and a gauge length of 25 mm were statically tested with the use of an upgraded hydraulic testing machine (installation) ZD-100Pu. When cutting test specimens, the fact that the pipes of the main gas pipelines are in different conditions of operation depending on the proximity to the pumping station was taken into account [17]. At the same time, the processes of aging and the accumulation of defects in the metal of pipes experiencing different power effects will occur in different ways. In this paper, the cutting of fragments (and, accordingly, specimens from them) was performed at a distance of less than 1/3 of the length of areas between pumping stations, which makes it possible to consider the studied specimens as the most damaged ones, since a higher level of operating pressure drops in pipelines at the outlet of pumping stations increases the average level of stress in the pipe wall [18]. The failure kinetics of the exploited steels of gas mains were investigated by the method of complete diagrams. The *K_λ_* parameter was determined, which is based on the concept of complete stress–strain diagrams of ductile materials and is adopted as a fast technique for evaluating the cracking strength [16].

(1) $$K_{\lambda }=\sqrt{S_{k}\cdot {\bar{\Delta l}}_{p}\cdot E}$$

here, *S_k_* is the actual resistance of the material to tearing; $\bar{\Delta l_{p}}$ is the specimen elongation at the growth stage of the separating macrocrack normalized to the cross-sectional area of the standard specimen; *E* is the Young’s modulus of materials.

The *K_λ_* criterion is an analog of the force criterion in fracture toughness test *K_IC_* and is related to the following dependence [16]:

(2) $$K_{IC}=\alpha K_{\lambda }$$

here, α is the proportionality factor (for steels it is equal to 0.23) [16].

Test specimens were ground and polished with the use of a Buehler Vector Phoenix Beta machine. The microstructure was analyzed after specimen etching in a 5% solution of nitric acid in ethyl alcohol. Analysis of chemical composition of pipe steels and their transitional phases was carried out using the Energy-dispersive X-ray spectroscopy (EDS) at JEOL JSM-35 CF as well as a LECO analyzer.

## 3. Degradation Processes in Gas Main Steels

**Figure 1:**
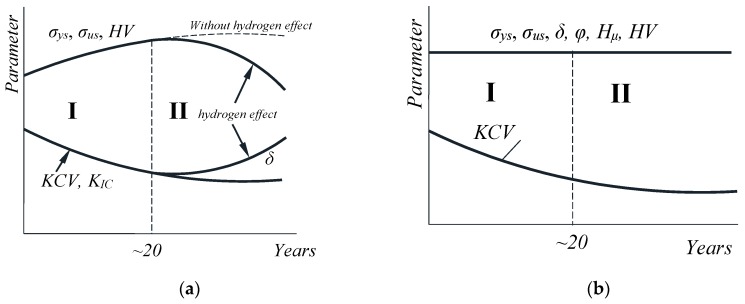
A generalized scheme of the gas main steel degradation: (**a**) In moderate climate conditions (Ukraine) [8]; (**b**) in conditions of the Far North (Yakutia, Russia) [12]; *KCV*—impact toughness; *K_IC_*—fracture toughness; *σ_us_*—ultimate strength; *σ_ys_*—yield stress; *HV*—hardness; *H_µ_*—microhardness; *δ*—relative elongation; *φ—*relative narrowing.

From the results of numerous literature data [19,20,21,22] and following the results of our previous investigations of gas pipeline steels from Ukraine [15,23,24,25], one of the main reasons for the degradation of gas main pipes is related to their strain aging. It gives rise to enhanced strength and decreased ductility, see stage I in Figure 1a.

Upon increasing the service life up to 20–30 years, the steel experiences volumetric damaging (formation of micropores). The latter results in some variations of mechanical properties of the pipe steels, see stage II in Figure 1a. The most critical among them is governed by the fact that the material hardening was almost leveled by the strain aging. In some cases, this was accompanied by a simultaneous decrease in strength, hardness, and resistance to brittle fracture.

The degradation processes in 09Mn2Si steel of the “Kysyl-Syr-Mastakh-Yakutsk” gas main (Russia, Yakutia) operated in Far North climatic conditions did result in a minor variation in the mechanical properties [12]. In doing so, regardless of the slight increase of the latter during the long-term operation, basic mechanical properties are illustrated by a straight line on the graph, since they did not go beyond the limits recommended by the industrial standards, see Figure 1b.

Note that the proposed degradation schemes are rather empirical since they are based on the synthesis of experimental data. Despite the fact that they cover most of the possible physical–mechanical states of the pipe steels, the particular duration of the degradation stages may vary. It should be stressed that the exact peculiarities of degradation processes are determined by (i) climatic conditions, (ii) stress–strain state of the pipe section under analysis; (iii) the isolation condition of the gas main as an additional factor.

## 4. Results and Discussion

### 4.1. Microstructure Analysis

**Figure 2:**
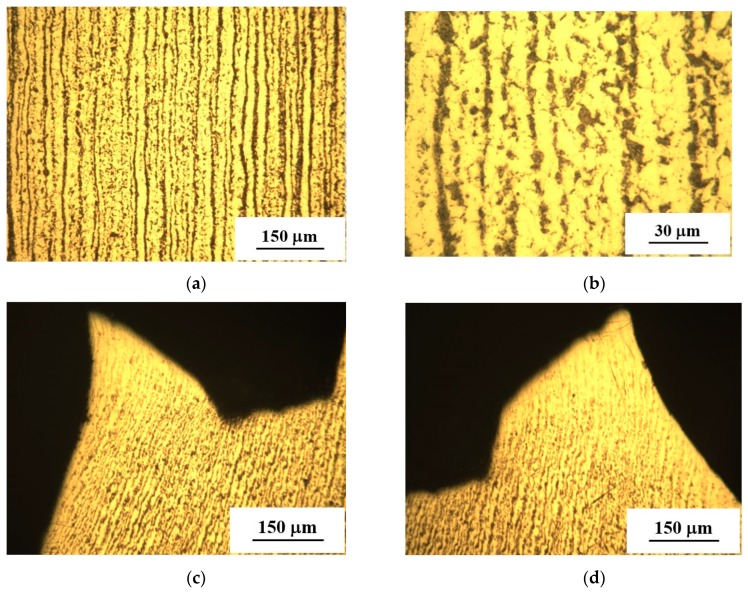
Microstructure of the fragment under investigation taken from the “Novopskov-Aksai-Mozdok” gas pipeline (Nevinnomysskaya CS): ferrite-pearlite structure with a striped morphology—(**a**) (×100); (**b**) (×400); microstructure in the neck region of tensile stretched specimens (×100) (**c**,**d**).

During long-term exploitation of structural steels, the combined effect of the stresses and active environmental medium (i.e., the hydrogen-containing one) upon the components of oil and gas infrastructure, in particular, the pipelines, are responsible for structural-phase transformations there [26]. Figure 2a,b shows the steel structure of the “Novopskov-Aksay-Mozdok” gas pipeline (Nevinnomysskaya CS) after long-term operation. It can be seen that cracks and stratifications can hardly be detected. The steel microstructure is represented by ferrite (light regions) and perlite (dark regions) phases. Steel exhibits a striped morphology since ferrite and perlite are arranged in the form of alternating stripes. The banded structure in the rolling direction of the sheet metal characteristic of the pipe manufacturing technology used in the 1980s creates conditions for strain aging in the metal, leading to the localization of microplastic strains and an increased risk of local “peaks” of stress that appear in the metal at the micro level. As a result, during the operation of pipes, the possibility of the local stress relaxation at the crack tip decreases, leading to an increased propensity of steels to brittle fracture. Pronounced strain localization is clearly seen in the neck region of the failed specimen, see Figure 2c,d. The fracture profile exhibits a branching pattern that indicates a pronounced efficiency of fracture energy [27]. Even at high strains, no laminations or cracks are formed along the direction of the structural strips. This additionally confirms the sufficiently high strength and ductility of the pipe steel.

**Figure 3:**
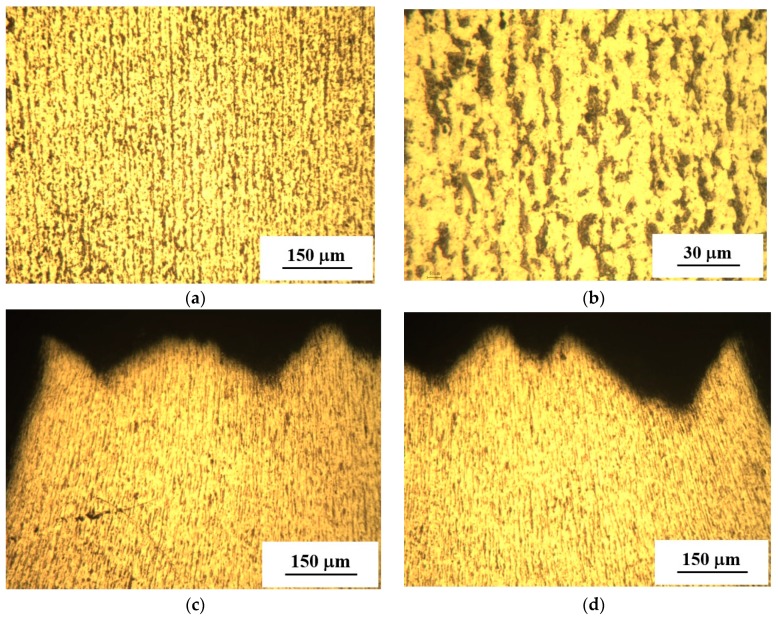
Microstructure of the fragment under investigation taken from the “Gorky-Center” gas pipeline steel (Gavrilovskaya CS): ferrite-perlite structure with striped morphology (**a**,**b**); microstructure of the tensile failed specimen in the neck region (**c**,**d**).

Figure 3a,b illustrates the structure of the “Gorky-Center” gas pipeline (Gavrilovskaya CS). It can be seen that the “strips” possess a broken profile, while the ferrite has a coarse-grained structure. A certain decrease in the perlite component amount in the vicinity of the outer surface of the pipe was found. The reason for the latter may be related to decarbonation [9]. If this take place, microstructural defects in the steel (complex alloyed carbides along the grain boundaries, nonmetallic inclusions having no adhesion bonding with the matrix, etc.) may be responsible for microcrack nucleation during the pipeline overloads. These will ease steel damaging at the substructural and structural scales. Much like the previous case, the zone of specimen failure, Figure 3c,d, is plastically deformed. Along with the absence of stratifications these testify for the sufficient ductility of the steel under analysis.

### 4.2. Complete Diagrams of Static Failure

**Figure 4:**
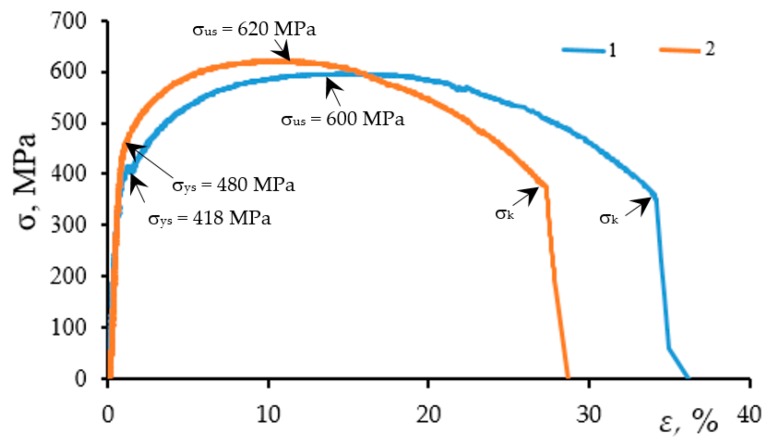
Complete stress–strain diagrams for steel specimens taken from the gas pipelines: 1—“Novopskov-Aksay-Mozdok” (Nevinnomysskaya CS); 2—“Gorky-Center” (Gavrilovskaya CS); *σ_k_*—is the stress acting on the specimen at the start of propagation of the macrocrack.

To evaluate the strength properties of the steels under study, the static stress–strain diagrams for the specimens were constructed, see Figure 4. Below, the main differences between the shape and parameters of the obtained diagrams are described.

The diagram for the steel taken from the “Novopskov-Aksai-Mozdok” gas pipeline (Nevinnomysskaya CS) is characteristic of ductile materials; thus, it is particularly pronounced at the yielding plateau stage. The form of the diagram is shallow; it is observed that the steel possesses sufficiently high ductility while maintaining high strength. The main crack starts to grow at a strain of 34%. From the chemical composition this steel is close to the 14Mn2 steel (Fe 98.25%, C 0.131%, Si 0.331%, Mn 1.148%, Cu 0.025%, Al 0.019%, Cr 0.052%, Mo 0.006%). At the same time, its key mechanical properties *σ_us_* = 600 MPa and *σ_ys_* = 418 MPa are much higher than those specified by the standard for this steel (*σ_us_* = 480 MPa and *σ_ys_* = 325 MPa, respectively).

From the chemical composition, the steel of the “Gorky-Center” gas main is close to that of the 17Mn1Si steel (Fe 97.25%; C 0.176%; Si 0.230%; Mn 0.931%; P,S < 0.002%; Cu 0.187%; Cr 0.54%; Mo 0.439%; Ni 0.156%). However, this steel possesses a greater tendency to strain hardening while the yield plateau can hardly be found. The propagation of the main crack starts at a strain of 26%. On the other hand, the fracture process (macrocrack propagation) runs slower compared to the above-described steel. Similarly to the previous case, the key mechanical properties *σ_us_* = 620 MPa and *σ_ys_* = 480 MPa are somewhat lower than those for the reserve steel (*σ_us_* = 660 MPa and *σ_ys_* = 570 MPa [28]), but higher than those specified by the standard requirements (GOST 19282-73—*σ_us_* = 510 MPa and *σ_ys_* = 345 MPa). 

Note that the mechanical properties of the specimens cut from the exploited pipes meet the requirements of the standards given for gas mains. However, it is suggested that high values of the yield strength and ultimate strength being of particular practical importance have resulted from the strain aging of the steels during operation. The parameters of the static cracking resistance calculated by the method of complete stress–strain diagrams [16] are summarized in Table 2.

### 4.3. Fractographic Research

**Figure 5:**
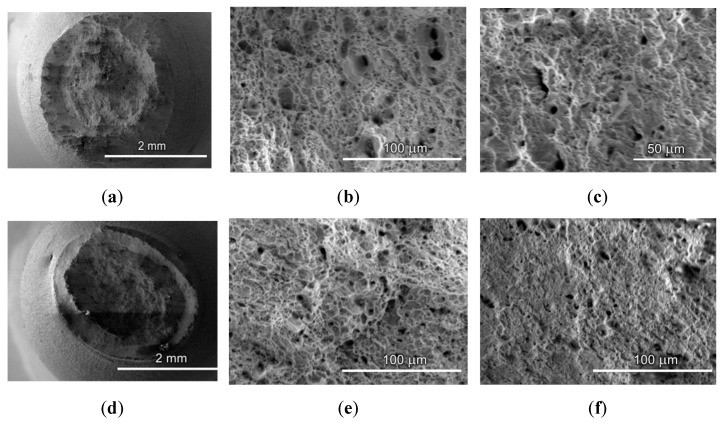
SEM micrographs of the fracture surface of the failed specimens taken from gas pipelines: (**a**–**c**) “Novopskov-Aksai-Mozdok” (Nevinnomysskaya CS); (**d**–**f**) “Gorky-Center” (Gavrilovskaya CS).

The fracture surface micrographs taken in the central part of the failed specimens were analyzed, see Figure 5. A large number of dimples of ductile separation can be seen. Also, the fracture surface is covered with a dense network of deep and large (2.0–10.0 μm) dimples. This can also be associated with the damage accumulation which occurred even during the operation time. The nucleation and coalescence of the pores takes place and is induced by the material segments displacements, as well as the formation and propagation of the main crack. At the fracture stage, the interstices between the pores decay the ability to uniform plastic flow. In doing so, strain localization is responsible for the formation of dimples on the fracture surface. Note that the fracture pattern of both the steels under investigation is uneven, which results from the fibrous structure and high anisotropy of the properties of the pipe steel, which is additionally enhanced due to the degradation [29].

**Figure 6:**
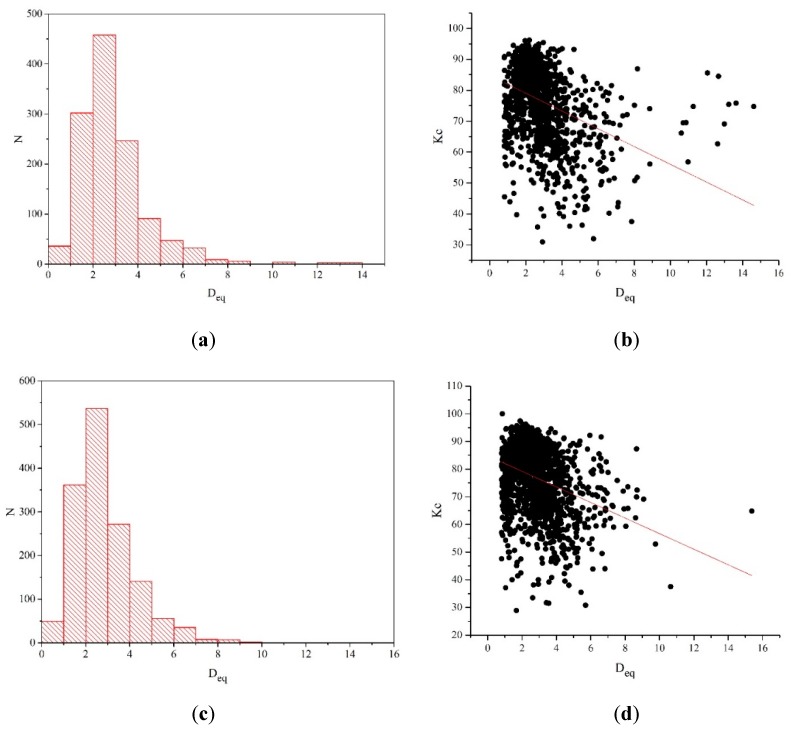
Distribution histograms on the diameters and shape coefficients of the dimples found on the steel fracture surface of the gas pipelines: (**a**,**b**) “Novopskov-Aksai-Mozdok” (Nevinnomysskaya CS); (**c**,**d**) “Gorky-Center” (Gavrilovskaya CS).

The dimple arrays on the fracture surface were analyzed with the use of an image processing algorithm (computer programs). The latter contains the operations of thresholding and filtering (with a set of special filters), as well as the analysis of bound domains [30]. The result of the ductile separation dimple analysis on the fracture surface is shown in Figure 6. Among the variables under calculation are:

(i) the equivalent diameter (*D_eq_*), see Figure 6а,c, that characterizes the dimple size, i.e., the diameter of the circle whose area is equal that of a dimple;
(ii) the shape coefficient *K_c_*, see Figure 6b,d, describes the similarity degree (level of approximation) of a dimple shape to a circular one. It characterizes the percentage of pixels that fall into a circle with a diameter *D_eq_* whose center coincides with the center of gravity of a dimple.

It was established that the separation dimple arrays for both specimens are very similar regarding the structure and shape.

However, dimensions of the individual dimples are quite different; larger dimples are surrounded by smaller ones. The dispersion graphs on their dimensions, see Figure 6a,d, indicate the heterogeneity of the material structure, which results from the non-uniformity of the plastic deformation development there [31]. The long-term exploitation, primarily in the case of hydrogenation, somewhat modifies the process of the microscale fracture of the steels under analysis. In doing so, the pore coalescence by ductile mechanism is alternated with microregions of spalling and quasi-separation by a mixed mechanism.

For both the investigated steels, it was found that the largest group consists of dimples with a diameter of 2–3 µm. The total number of dimples is 450–550. The maximum dimple diameter is equal to 10–14 µm. High values of the shape coefficient (>60% for the majority of dimples) confirm he circular shape of the dimples. Upon increasing their size, the interaction between the adjacent dimples and their boundaries becomes closer. The latter is responsible for their elongated shape. The fitting line, see Figure 6b,d, is inclined by the same angle for both specimens (at 70.4° and 70.8°, respectively). This indicates that the dimples revealed on the fracture surfaces of both steel specimens have a similar shape.

In doing so, the data of the fractographic studies allowed us to reveal that during the gas main steel exploitation certain type of defects nucleate which are not characteristic of the materials’ initial state. However, the numerical analysis on the dimples’ shape and size found on the fracture surface made it possible to characterize their “scattered” nature, with the nearly absent or insignificant interactions between the adjacent defects [32,33,34,35]. This ensures higher durability, ductility and crack resistance of the investigated pipe steels. The latter result explains the detected anomalies in the mechanical properties variation for the steels after long-term exploitation. The additional degradation mechanism is related to the strain aging, i.e., the increase in the dislocation density, and, consequently, in the strength of the steels under investigation.

## 5. Conclusions

The characteristic features of the steel degradation in the “Novopskov-Aksai-Mozdok” gas pipeline (Nevinnomysskaya CS) and the “Gorky-Center” gas pipeline (Gavrilovskaya CS) can be treated as a particular case of damage accumulation by the scheme proposed by Prof. H.M. Nykyforchyn. However, in contrast to the “classical” case, section I of the diagrams is elongated, while the mechanical state of these steels can be interpreted as a transient condition from the damage Stage I to Stage II.

It has been experimentally established that the strength, ductility and cracking resistance properties of the investigated steels are quite high. Their in-service hydrogenation is accompanied by the microplastic deformation and nucleation of micropores due to static deformation. However, the pore formation mechanism was localized due to “scattered” damaging. 

Both types of zones of the ductile separation and individual regions of micro-spallation were found on the fracture surface. These testify to the embrittlement effect induced by the hydrogen absorption. In general, the steels under investigation possess a sufficiently high level of mechanical properties required for further safe exploitation, both in terms of durability and cracking resistance. 

## Figures and Tables

**Table 1:** Metal exploitation parameters of the gas pipelines.

Gas Pipeline, Compressor Station	Working Pressure, atm	Pipe Diameter, mm	Run Time, years
“Gorky-Center”, Gavrilovskaya CS	55	1020	39
“Novopskov-Aksay-Mozdok”, Nevinnomysskaya CS			30

**Table 2:** Cracking resistance parameters *K_λ_* and *K_IC_* for the steel after a long-term operation.

Gas Pipeline, Compressor Station	Experimental Data			Results Calculated by the Equations (1) and (2)	
	$\bar{\Delta l_{p}},\;\mathrm{mm}$	F_k_ *, kN	*E*, GPa	*K_λ_*, ${\mathrm{MPa}}^{\sqrt{m}}$	*K_IC_*, ${\mathrm{MPa}}^{\sqrt{m}}$
“Gorky-Center”, (Gavrilovskaya CS)	0.206	6.537	1.7	471.1	108.353
“Novopskov-Aksay-Mozdok”, (Nevinnomysskaya CS)	0.266	5.705	1.7	358.9	82.547

* *F_k_*—is the force acting on the specimen at the start of propagation of the macrocrack [16].
